# PRISM-games: verification and strategy synthesis for stochastic multi-player games with multiple objectives

**DOI:** 10.1007/s10009-017-0476-z

**Published:** 2017-11-29

**Authors:** Marta Kwiatkowska, David Parker, Clemens Wiltsche

**Affiliations:** 1 Department of Computer Science, University of Oxford, Oxford, UK; 2 School of Computer Science, University of Birmingham, Birmingham, UK

**Keywords:** Formal verification, Quantitative verification, Stochastic games

## Abstract

PRISM-games is a tool for modelling, verification and strategy synthesis for stochastic multi-player games. These allow models to incorporate both probability, to represent uncertainty, unreliability or randomisation, and game-theoretic aspects, for systems where different entities have opposing objectives. Applications include autonomous transport, security protocols, energy management systems and many more. We provide a detailed overview of the PRISM-games tool, including its modelling and property specification formalisms, and its underlying architecture and implementation. In particular, we discuss some of its key features, which include multi-objective and compositional approaches to verification and strategy synthesis. We also discuss the scalability and efficiency of the tool and give an overview of some of the case studies to which it has been applied.

## Introduction

Automatic verification and strategy synthesis are techniques for analysing probabilistic systems. They can be used to produce formal guarantees with respect to quantitative properties such as safety, reliability and efficiency. For example, they can be employed to synthesise controllers in applications such as autonomous vehicles, network protocols and robotic systems. These often operate in uncertain and adverse environments, models of which require both stochasticity, for example, to represent noise, failures or delays, and game-theoretic aspects, to model non-cooperative agents or uncontrollable events.

PRISM-games is a tool for verification and strategy synthesis for turn-based *stochastic multi-player games*, a model in which each state is controlled by one of a set of players. That player resolves non-determinism in its states by selecting an action to perform. The resulting behaviour, i.e. to which state the model then evolves, is probabilistic. This allows the model to capture both game-theoretic aspects and stochasticity.

The crucial ingredient for reasoning about stochastic multi-player games is *strategies*, which represent the choices made by a given player, based on the execution of the model so far. For a stochastic game comprising just one player (in other words, a Markov decision process), we may choose to consider the behaviour of the player to be adversarial (for example, representing the malicious environment of a security protocol). We can then *verify* that the model exhibits certain formally specified properties, regardless of the behaviour of the adversary.

Alternatively, we could assume that we are able to control the choices of the single player in this model (imagine, for example, it represents the navigation control system in an autonomous vehicle). In this setting, we can instead use *strategy synthesis* to generate a strategy (a controller) under which the behaviour of the game satisfies a formally specified property.

The general case, in which there are multiple players, allows us to model situations where there are entities with opposing objectives, for example a controller *and* a malicious environment. PRISM-games provides strategy synthesis techniques that can generate a strategy for one player of a stochastic game such that it is guaranteed to satisfy a property, regardless of the strategies employed by the other players. Returning to the autonomous vehicle above, we could generate a strategy for the vehicle controller which guarantees that the probability of successfully completing a journey is above a specified threshold, regardless of the behaviour of other, uncontrollable aspects of the system such as other road users.

This paper provides an overview of PRISM-games and the strategy synthesis techniques that it provides. These fall into two categories. The first, *single-objective* case, is used to express zero-sum properties in which two opposing sets of players aim to minimise and maximise a single objective: either the probability of an event or the expected reward accumulated before it occurs. The second, *multi-objective* case, enables the exploration of trade-offs, such as between performance and resource requirements. The tool also performs computation and visualisation of the *Pareto sets* representing the optimal achievable trade-offs.

We also discuss the support in PRISM-games for *compositional* system development. This is done through *assume-guarantee* strategy synthesis, based on contracts over component interfaces that ensure cooperation between the components to achieve a common goal. For example, if one component satisfies the goal *B* under an assumption *A* on its environment (i.e. $A \rightarrow B$), while the other component ensures that the assumption *A* is satisfied, we can compose strategies for the components into a strategy for the full system achieving *B*. Multi-objective strategy synthesis, e.g. for an implication $A \rightarrow B$, can be conveniently employed to realise such assume-guarantee contracts. Again, Pareto set computation can be performed to visualise the relationship between properties and across interfaces.

The underlying verification and strategy synthesis techniques developed for PRISM-games have been published elsewhere, in [5, 7, 12, 14, 16, 44]. Existing short tool papers focusing on the functionality added in versions 1.0 and 2.0 of PRISM-games were presented in [13] and [34], respectively. This paper provides a comprehensive overview of the full tool, including detailed examples of the modelling and property specification and summaries of the key theory and algorithms. We also discuss implementation details, the scalability of the tool and the application domains to which it has been applied.

**Structure of the paper** Section  2 provides basic details of the underlying model of stochastic multi-player games and explains how these can be described using the PRISM-games modelling language. Section 3 covers the property specification language, giving the formal syntax, semantics and examples of the various classes of quantitative properties that are supported. Section 4 gives an overview of the underlying algorithms used to perform verification and strategy synthesis, and Sect. 5 describes the architecture of the tool and some lower-level aspects of its implementation. Section 6 presents some experimental results and discusses the scalability and efficiency of PRISM-games. We conclude, in Sects. 7, 8 and 9, with a discussion of case studies to which the tool has been applied, a survey of related tools and some areas of current and future work.

## Models and modelling

We begin by explaining the underlying models used by PRISM-games and the means by which they are specified to the tool. We will use ${{ Dist }}(S)$ to denote the set of discrete probability distributions over a set *S*.

### Stochastic multi-player games

The primary probabilistic model supported by PRISM-games is *stochastic multi-player games* (SMGs). These model systems whose evolution is determined by the decisions of multiple *players* plus the presence of probabilistic behaviour. We restrict our attention here to *turn-based* (as opposed to *concurrent*) stochastic games, in which a single player controls each state of the model.

#### Definition 1

(*SMG*) A *stochastic multi-player game* (SMG) is a tuple $\mathcal {G}= (\varPi ,S,(S_i)_{i\in \varPi },{\overline{s}},A,\delta ,L)$, where:

- $\varPi$ is a finite set of *players*,
- *S* is a finite set of *states*,
- $(S_i)_{i\in \varPi }$ is a partition of *S*,
- ${\overline{s}}\in S$ is an initial state,
- $A$ is a finite set of *actions*,
- $\delta : S {\times } A\rightarrow {{ Dist }}(S)$ is a (partial) probabilistic transition function,
- $L:S\rightarrow 2^{ AP }$ is a labelling function mapping states to sets of atomic propositions from a set ${ AP }$.

The state of an SMG $\mathcal {G}$ is initially ${\overline{s}}$, and it then evolves as follows. In any state *s*, there is a non-deterministic choice between the set of enabled actions $A(s)\subseteq A$, where . We assume that $A(s)$ is non-empty for all states *s*, i.e. that there are no deadlock states in the model. (This is checked and enforced by the tool.) The choice of an action from $A(s)$ is resolved by the player that *controls* the state *s*, i.e. the unique player $i\in \varPi$ for which $s\in S_i$. Once this player selects an action $a\in A$, a transition to a successor state $s'$ occurs randomly, according to the probability distribution $\delta (s,a)$, i.e. the probability that a transition to $s'$ occurs from the current state *s* is $\delta (s,a)(s')$.

A *path* through $\mathcal {G}$, representing one possible execution of the system that it models, is a (finite or infinite) sequence of states and actions $\pi =s_0 a_0 s_1 a_1 s_2 \ldots$, where $s_i\in S$, $a_i\in A(s_i)$ and $\delta (s_i,a_i)(s_{i+1}){>}0$ for all $i \in \mathbb {N}$. We write $FPath _{\mathcal {G},s}$ and $IPath _{\mathcal {G},s}$, respectively, for the set of all finite and infinite paths of $\mathcal {G}$ starting in state *s* and denote by $FPath _\mathcal {G}$ and $IPath _\mathcal {G}$ the sets of *all* such paths.

To reason about the various possible ways in which the game can behave, we use the notion of *strategies*, which define the choices of actions made by players in each state, based on the history of the game’s execution so far. Formally, these are defined as follows.

#### Definition 2

(*Strategy*) A *strategy* for player $i\in \varPi$ in SMG $\mathcal {G}$ is a function ${\sigma }_i: (SA)^*S_i \rightarrow {{ Dist }}(A)$ which, for each path $\lambda {\cdot }s\in FPath _{\mathcal {G}}$ where $s\in S_i$, selects a probability distribution ${\sigma }_i(\lambda {\cdot }s)$ over $A(s)$. The set of all strategies for player *i* is denoted ${\varSigma }_i$.

Various important classes of strategies can be identified, by classifying the extent to which they use the history of the game’s execution so far and whether or not they use randomisation. For the latter, a strategy is called *deterministic* (or pure) if the distribution used to select actions is always a point distribution (i.e. selects a single action with probability 1), and *randomised* otherwise. Regarding the use of history (i.e. *memory*), we identify the following important classes.

#### Definition 3

(*Memoryless strategy*) A strategy ${\sigma }_i$ is called *memoryless* if it only considers the current state when resolving non-determinism, i.e. if ${\sigma }_i(\lambda {\cdot }s)={\sigma }_i(\lambda '{\cdot }s)$ for all paths $\lambda {\cdot }s,\lambda '{\cdot }s\in FPath _{\mathcal {G}}$.

A *finite-memory* strategy has a *mode*, which is updated each time a transition is taken in the game and then used to select an action in each state.

#### Definition 4

(*Finite-memory strategy*) A *finite-memory* strategy is defined by a tuple $(Q,q_0,{\sigma }_i^u,{\sigma }_i^s)$ comprising:

- a finite set of *modes* (or *memory elements*) *Q*;
- an initial mode $q_0\in Q$;
- a mode update function ${\sigma }_i^u:Q {\times } S_i \rightarrow { Dist }(Q)$;
- an action selection function ${\sigma }_i^s:Q {\times } S_i \rightarrow { Dist }(A)$

The mode *q* of the strategy initially takes the value $q_0$ and is updated according to the distribution ${\sigma }_i^u(q,s)$ for each observed state *s* of the game $\mathcal {G}$. At each step of the game’s execution, if the mode is *q* and the current state of $\mathcal {G}$ is *s*, then the strategy chooses an action according to the distribution ${\sigma }_i^s(q,s)$.

In addition to the distinction mentioned above between randomised and deterministic strategies, finite-memory strategies are classified as *deterministic memory update* strategies if the mode update function ${\sigma }_i^u$ always gives a point distribution over modes, and *stochastic memory update* strategies otherwise.

Given multiple strategies ${\sigma }_i\in {\varSigma }_i$ for several players $i\in \varPi$, we can combine them into a single strategy that resolves choices in all the states controlled by those players. For example, for strategies ${\sigma }_1$ and ${\sigma }_2$ for players 1 and 2, we write ${\sigma }=({\sigma }_1,{\sigma }_2)$ to denote the combined strategy $\sigma$. For a *coalition* of players $C\subseteq \varPi$, we use $\varSigma _C$ to denote the set of all (combined) strategies for players in *C*. If a strategy $\sigma$ comprises strategies for all players of the game (sometimes called a *strategy profile*), we can construct a probability space ${ Pr _{{\mathcal {G}}}^{\sigma }}$ over the infinite paths of ${\mathcal {G}}$. This measure also allows us to define the *expectation* $\mathbb {E}_{\mathcal {G}}^{\sigma }[\rho ]$ of a measurable function $\rho$ over infinite paths. We use ${ Pr _{{\mathcal {G}}}^{\sigma }}$ and $\mathbb {E}_{\mathcal {G}}^{\sigma }[\rho ]$ to formally define a variety of quantitative properties of a game’s behaviour under a particular strategy, notably the probability of some measurable event, or the expected value of a reward/cost measure, respectively.

As shown in Definition 1, states of SMGs are labelled with atomic propositions, from some set ${ AP }$. These are used to identify particular states of interest when writing temporal logic formulas to describe an event (see Sect. 3). We also augment SMGs with *reward structures* of the form $r:S\rightarrow \mathbb {R}_{\geqslant 0}$, which assign non-negative, real-valued rewards to the states of a game. These can have many different interpretations and are, in fact, often used to capture *costs* rather than rewards (for example, energy usage or elapsed time).

### Subclasses of SMGs

Next, we identify some useful subclasses of stochastic multi-player games, which may be simpler to analyse, or be amenable to verification against a wider range of properties than the general model.

**Stochastic two-player games** Firstly, we observe that, when the cardinality of the player set $\varPi$ of an SMG is 2, the SMG is a (turn-based) *stochastic two-player game* [37], a widely studied class of models, sometimes also known as $2\frac{1}{2}$-player games.

In practice, these suffice for modelling many real-life scenarios in which there is a natural separation into two competing entities (for example, defender vs. attacker in the context of a security protocol, or controller vs. environment in the context of a control problem). In fact, for the properties currently supported by PRISM-games, the verification of stochastic *multi-player* games actually reduces to the problem of solving one or more stochastic two-player games.

**Markov decision processes** It is also worth noting that, when an SMG contains only one player (or, when all but one player has a singleton set of choices in all of their states), the SMG is in fact a *Markov decision process* (MDP), sometime also called a $1\frac{1}{2}$-player game. The basic problem of strategy synthesis for MDPs has a lower time complexity than for SMGs (polynomial, rather than NP $\cap$ coNP), and support for this model is already implemented in the regular version of PRISM.

**Stopping games** When we discuss multi-objective techniques for SMGs later in the paper, we will refer to two subclasses of models for which additional property classes are available. The first are *stopping games*. We call states in an SMG from which no other state can be reached under any strategy *terminal states*. A stopping game is an SMG in which terminal states are reached with probability 1 under any strategy.

**Controllable multi-chain games** The second subclass of SMGs relevant for multi-objective properties are *controllable multi-chain* games [7]. Intuitively, an SMG is controllable multi-chain if one player (or set of players) has the ability to control which subset of states the game eventually reaches and remains within. The formal definition relies on a generalisation of the notion of *end components* for MDPs [20] to *irreducible components* [7], which are strongly connected fragments of the SMG that, once reached, will never be left. Controllable multi-chain means that one player can, for each irreducible component *H*, and starting in any state *s* of the SMG, ensure that *H* is reached from *s* with probability 1. We refer the reader to [7] for a full definition.

### Modelling SMGs in PRISM-games

PRISM-games extends the existing PRISM modelling language to provide a formalism for specifying SMGs to be analysed by the tool. In this section, we explain the language and illustrate it using an example.

PRISM uses a textual modelling language, originally inspired by the Reactive Modules formalism of [1], and based on guarded command notation. A model comprises one or more *modules* whose state is determined by a set of *variables* and whose behaviour is defined by a set of *guarded commands* of the form:

$$\begin{aligned} {\texttt {[act] guard -> p}_{\texttt {1}}{\texttt { : update}_{\texttt {1}}{\texttt { + ... + p}_{\texttt {n}}{\texttt { : update}_{\texttt {n}};}}}} \end{aligned}$$

This comprises an (optional) action label $\texttt {act}$, a *guard* (a predicate over the variables of all modules in the model), and a list of *updates*, with associated probabilities. Each update $\texttt {update}_{\texttt {i}}$ is a list of assignments $\texttt {(v}_{\texttt {j}}{} \texttt {'=expr}_{\texttt {j}})$ which, if executed, would evaluate the expression $\texttt {expr}_{\texttt {j}}$ and assign the value to variable $\texttt {v}_{\texttt {j}}$. Each $\texttt {p}_{\texttt {i}}$ is an expression over variables of the model which, when evaluated, gives the probability for each update. Intuitively, when a module has a command whose guard is satisfied in the current state, that module can update its variables probabilistically, according to the updates.

**Fig. 1:**
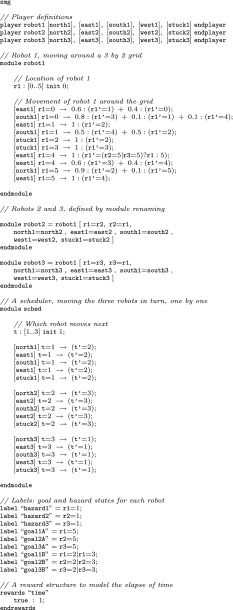
A PRISM-games description of a three-player SMG modelling 3 robots navigating around a 3$\times$2 grid

**Fig. 2:**
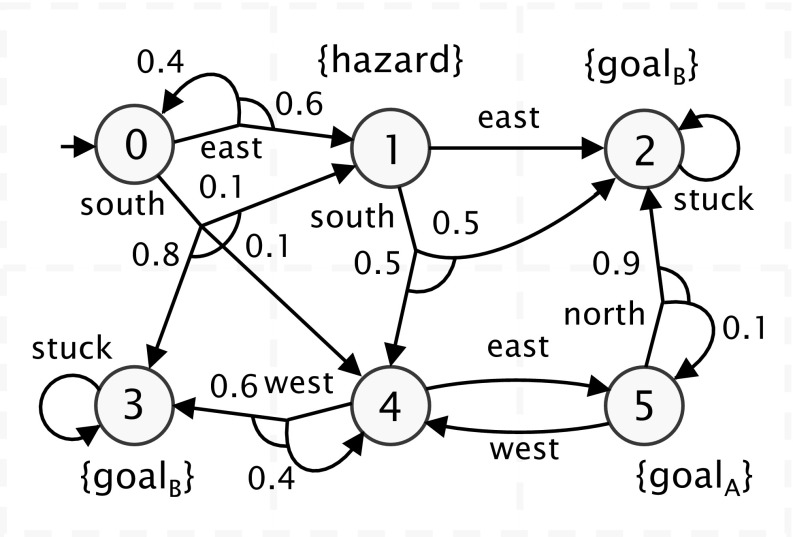
Probabilistic movement of a robot around a 3$\times$2 grid; used for the example model of Sect. 2.3 (see Fig. 1)

Action labels serve several purposes. First, they are used to annotate the transitions in the SMG, i.e. they represent the actions in the set $A(s)$ for each state *s* that are chosen by the players. Secondly, they provide a means for multiple modules to execute their updates synchronously. More formally, this is done using a multi-way parallel composition of processes, which is based on the definition of parallel composition proposed by Segala for probabilistic automata [36]. Lastly, we also use action labels to specify which parts of the model belong to which player. This is explained below, using an example.

**Example** Figure 1 shows a PRISM-games model of an SMG representing three robots navigating a space that is divided into a $3\times 2$ grid. The grid, and the movements that can be made around it, is shown in Fig. 2. A robot can select a direction of movement in each grid location ($north$, $east$, etc.) but, due to the possibility of obstacles, may with some probability end up remaining in the same location or moving to a different one.

The first module shown in Fig. 1 represents robot 1, whose variable r1 gives its current grid location (using the numbering from Fig. 2). The guarded commands represent the possible actions that can be taken and the resulting probabilistic updates to variable r1. The second and third modules, for robots 2 and 3, are identical to the one for robot 1, except that the states of those robots are represented by different variables (r2 and r3), and the action labels are different (e.g. north2 and north3, rather than north1). So, these modules are defined using PRISM’s *module renaming* feature.

The actions of the robots are mostly independent; however, no two can be in location 5 simultaneously. Hence, the update corresponding to a move into this location is written as (r1’=(r2=5|r3=5)?r1:5), which means that r1 is updated to 5 only if r2 or r3 is not already equal to 5; otherwise it remains unchanged.

We also comment on the use of parallel composition in the model. PRISM-games currently only supports turn-based SMGs. As a simple way of ensuring this is respected for the example model, we make the robots move in sequence, one by one. To enforce this, we make the action labels for modules robot1, robot2 and robot3 disjoint and add a fourth module sched, with a variable t denoting the robot who is next to move and which synchronises with the appropriate module, depending on the current value of t. This is a relatively common approach in PRISM-games models where the concurrency between parallel components is controlled to allow it to be represented by a turn-based SMG.

The specification of the players in the SMG can be seen near the top of Fig. 1. This defines the set of players in the SMG, and their names, and then assigns to each one a set of action names. This effectively associates each transition in the SMG with a particular player. Currently, since PRISM-games only supports turn-based games, it checks each state to ensure that all actions from that state are associated with the same player and then assigns the state to that player. This approach (identifying player states via action names) is taken in order to provide compatibility with models such as concurrent SMGs, for which we plan to add support in future.

Finally, we note that the last part of the PRISM-games model contains some straightforward definitions of *labels* and *rewards*, which are done in the same way as for standard PRISM models. Labels are used to identify states of interest, for the purposes of property specification, and are defined by a predicate over state variables; for this example, they are used to identify when each robot is in a location marked as a $goal$ (either *A* or *B*) or a $hazard$ in Fig. 2. There is also a very simple reward structure $time$, modelling the elapse of time, defined by assigning a fixed reward of 1 to every state.

**Compositional modelling** Version 2.0 of the PRISM-games tool added support for compositional strategy synthesis using assume-guarantee proof rules (see Sect.  4.3 for more details). In order to facilitate this, the modelling language also provides a specific compositional modelling approach designed for assume-guarantee strategy synthesis. This works for stochastic *two-player* games, specifically targeting controller synthesis problems modelled with games where player 1 represents a controller and player 2 its environment.

It allows a game to be defined as several *subsystems*, each of which comprises a set of modules, which are combined using the normal parallel composition of PRISM-games. Subsystems are combined using the game composition operator introduced in [6]; actions controlled by player 1 in subsystems are also controlled by player 1 in the composition, thus enabling composition of the synthesised player 1 strategies. This allows controller synthesis problems to be solved in a compositional fashion. For more details of this aspect of the language, we refer the reader to [44].

## Property specification

In order to formally specify the desired behaviour of an SMG, we use *properties*. In PRISM-games, properties are specified as temporal logic formulas. As a basis for this, we use the core part of the existing property specification logic of PRISM [32], which is itself based on the probabilistic temporal logic PCTL [28], extended with operators to reason about rewards [25].

PRISM-games currently supports two main classes of properties: (i) single-objective and (ii) multi-objective. Although these fit within the same unified property specification language, there are different syntactic restrictions imposed in each case, so we present the two classes separately. In the explanations that follow, we will assume that we are specifying properties for a fixed SMG $$\mathcal {G}= (\varPi ,S,(S_i)_{i\in \varPi },{\overline{s}},A,\delta ,L)$$. When giving examples of such properties, we will continue to use the SMG illustrated in the previous section, which has 3 players, $$ robot _1$$, $$ robot _2$$ and $$ robot _3$$. Each label from Fig. 1 (e.g. “goal1A”) corresponds to an atomic proposition (e.g. $$ goal_{1A} $$), which can be used in temporal logic properties.

### Single-objective properties

For *single-objective* properties of SMGs, we use a fragment of the property specification language called rPATL (probabilistic alternating-time temporal logic with rewards), originally proposed in [12]. This adopts the coalition operator $\langle \!\langle \cdot \rangle \!\rangle$ from the logic ATL [2], used for reasoning about strategies in non-probabilistic games, and adds the probabilistic operator ${\mathtt P}$ from PCTL and an extension of PRISM’s reward operator ${\mathtt R}$ [25]. The formal definition is as follows.

#### Definition 5

(*rPATL syntax*) The syntax of the logic rPATL is given by the grammarwhere $a{\in } AP$, $C\subseteq \varPi$, $\bowtie \,{\in }\{<, \leqslant , \geqslant , >\}$, $p{\in } \mathbb {Q}\cap [0,1]$, $x {\in } \mathbb {Q}_{\geqslant 0}$, *r* is a reward structure, $\star {\in } \{0,\infty ,c\}$ and $k{\in }\mathbb {N}$.

A property of an SMG $\mathcal {G}$ expressed in rPATL is a formula from the rule $\phi$ in the syntax above. The key operator is $\langle \!\langle C \rangle \!\rangle \theta$, where $C\subseteq \varPi$ is a *coalition* of players from $\mathcal {G}$ and $\theta$ is a quantitative objective that this set of players will aim to satisfy. An objective $\theta$ is a single instance of either the ${\mathtt P}$ or ${\mathtt R}$ operator: ${\mathtt P}_{\bowtie \,p}[\,{\cdot }\,]$ means that the probability of some event being satisfied should meet the bound $\bowtie p$, and ${\mathtt R}^{r}_{\,\bowtie \,x}[\,{\cdot }\,]$ means that expected value of a specified reward measure (using reward structure *r*) meets the bound $\bowtie x$. For example, the property:

$$\begin{aligned} \langle \!\langle \{ robot _1\} \rangle \!\rangle {\mathtt P}_{\geqslant 0.75}[\,{\lnot hazard_1 {\ {\mathtt U}^{\leqslant 10}\ } goal_{1A} }\,] \end{aligned}$$

asserts that there exists a strategy for $$ robot _1$$ under which, regardless of the strategies chosen by the other players (in this case, $$ robot _2$$ and $$ robot _3$$), the probability of reaching a “goal *A*” state within 10 steps, without passing through a “hazard” state, is at least 0.75.

In general, the form of the probabilistic operator is ${\mathtt P}_{\bowtie \,p}[\,{\psi }\,]$, where the event whose probability is being referred to is specified as a *path formula* $\psi$. As shown in the grammar, we allow three basic types of path formulas, taken from PCTL:  (“next”: $\phi$ is true in the next state); $\phi _1{\ {\mathtt {U}}^{\leqslant k}\ }\phi _2$ (“bounded until”: $\phi _2$ is true within *k* steps and $\phi _1$ remains true in all states until that point); and $\phi _1{\ {\mathtt {U}}\ }\phi _2$ (“until”: $\phi _2$ is eventually true and $\phi _1$ remains true in all states until that point). As usual, we can derive other common temporal operators such as ${{{\mathtt {F}}}\ }\phi \equiv \mathtt {true}{\ {\mathtt {U}}\ }\phi$ (eventually $\phi$ is true) and ${{{\mathtt G}}\ }\phi \equiv \lnot {{{\mathtt {F}}}\ }\lnot \phi$ ($\phi$ always remains true forever). An example is:

- $\langle \!\langle \{ robot _1, robot _2\} \rangle \!\rangle {\mathtt P}_{\geqslant 0.9}[\,{{{{\mathtt {F}}}\ }( goal _{1B}{\wedge } goal _{2B})}\,]$ —robots 1 and 2 have strategies which ensure that, with probability at least 0.9, they are eventually both in a goal *B* state simultaneously, regardless of the actions taken by robot 3.

The reward operator ${\mathtt R}^{r}_{\,\bowtie \,x}[\,{{{{\mathtt {F}}}\ }\!\!^{\star }\phi }\,]$ allows us to reason about the expected amount of reward *r* accumulated until $\phi$ is true, i.e. the expected sum of rewards along a path until a state satisfying $\phi$ is first reached. The $\star$ parameter (which takes one of the values 0, $\infty$ or *c*) is used to indicate how to treat the situation where a $\phi$-state is *not* reached. When this occurs, the cumulated reward for that path is, for the three cases $\star = 0$, $\infty$ or *c*, taken to be zero, infinite or equal to the cumulated reward along the whole path, respectively.

These three different variants are provided because each has its own uses depending on the nature of the reward structure being used. Consider, for example, a situation where the goal of a player is to minimise the expected time for an algorithm to complete. In this case, it is natural to assume a value of infinity upon non-completion ($\star {=}\infty$). An alternative example, on the other hand, would be where we are trying to model and analyse a distributed algorithm by investigating a reward structure that incentivises certain kinds of behaviour, and aiming to maximise it over the lifetime of the algorithm’s execution. Then, parameter $\star {=}0$ might be preferred to avoid favouring situations where the algorithm does not terminate. Lastly, when modelling for example, an algorithm’s resource consumption, we might opt to use type $\star {=c}$, to represent resources used regardless of termination. An example of the first type would be:

- $\langle \!\langle \{ robot _1\} \rangle \!\rangle {\mathtt R}^{ time }_{\,\leqslant 10}[\,{{{{\mathtt {F}}}\ }\!\!^{\infty } goal _{1A}}\,]$ —it is possible for robot 1 to ensure that the expected time taken to reach goal *A* is at most 10.

Recall that the $time$ reward structure from the example SMG simply assigns a fixed reward of 1 to every state.

Notice also that the syntax of rPATL allows us to construct Boolean combinations of properties $\phi$ and that, further, instances of the $\langle \!\langle C \rangle \!\rangle \theta$ operator can be nested to create more complex formulas. Examples of each include:

- $\langle \!\langle \{ robot _1\} \rangle \!\rangle {\mathtt P}_{\geqslant 1}[\,{{{{\mathtt {F}}}\ } goal_{1B} }\,] \vee \langle \!\langle \{ robot _2\} \rangle \!\rangle {\mathtt P}_{\geqslant 1}[\,{{{{\mathtt {F}}}\ } goal_{2B} }\,]$ —at least one of robot 1 and robot 2 has a strategy to ensure it reaches goal *B* with probability 1;
- $\langle \!\langle \{ robot _1\} \rangle \!\rangle {\mathtt P}_{<0.01}[\,{{{{\mathtt {F}}}\ }\lnot \langle \!\langle \{ robot _3\} \rangle \!\rangle {\mathtt P}_{\geqslant 0.95}[\,{{{{\mathtt G}}\ }\lnot hazard_{3} }\,]}\,]$ —it is possible for robot 1 to ensure that, with probability less than 0.01, a state is reached from which robot 3 is unable to guarantee that it avoids hazard states with probability at least 0.95.

Another useful class of properties, which are not explicitly included in the syntax above, are *numerical* queries. For a property comprising a single $\langle \!\langle C \rangle \!\rangle \theta$ operator, the bound $\bowtie p$ or $\bowtie x$ in the ${\mathtt P}$ or ${\mathtt R}$ is replaced with either “min=?” or “max=?” and, rather than asking about the existence of a strategy satisfying the bound, yields the optimal (minimum or maximum) value obtainable by a strategy. Examples are:

- $\langle \!\langle \{ robot _1\} \rangle \!\rangle {\mathtt P}_{\max =?}[\,{{{{\mathtt G}}\ }\lnot hazard_1 }\,]$ —what is the maximum probability with which robot 1 can guarantee that it avoids $hazard$ states?
- $\langle \!\langle \{ robot _2\} \rangle \!\rangle {\mathtt R}^{ time }_{\,\min =?}[\,{{{{\mathtt {F}}}\ } goal _{2A}}\,]$ —what is the minimum expected time with which robot 2 can reach goal *A*?

### Multi-objective properties

An important class of properties, added in version 2.0 of PRISM-games, are those which characterise the goals of a player (or players) using *multiple objectives*. We continue to use the coalition operator $\langle \!\langle C \rangle \!\rangle \theta$ discussed above, but now allow $\theta$ to be, for example, a conjunction of different objectives all of which need to be satisfied. More generally, we allow $\theta$ to be a Boolean combination of different objectives. For this class of properties, we focus purely on reward-based properties and consider a different range of reward objectives, which we explain in more detail below. The full syntax is as follows.

#### Definition 6

(*Multi-objective syntax*) The syntax for multi-objective properties is given by the grammar:

$$\begin{aligned} \phi&{:}{:}= \mathtt {true}\mid a \mid \lnot \phi \mid \phi \wedge \phi \mid \langle \!\langle C \rangle \!\rangle \theta \\ \theta&{:}{:}= {\mathtt R}^{r}_{\,\bowtie x}[\,{\mathtt {C}}\,] \mid {\mathtt R}^{r}_{\,\bowtie x}[\,{{\mathtt S}}\,] \mid {\mathtt R}^{r/r}_{\,\bowtie x}[\,{{\mathtt S}}\,] \mid {\mathtt P}_{\geqslant 1}[\,{\psi }\,] \mid \!\lnot \!\theta \mid \theta \!\wedge \!\theta \\ \psi&{:}{:}= {\mathtt R}^{r}_{\,\bowtie x}[\,{{\mathtt S}}\,] \mid {\mathtt R}^{r/r}_{\,\bowtie x}[\,{{\mathtt S}}\,] \end{aligned}$$

where $a \in AP$, $C\subseteq \varPi$, $\bowtie \,{\in }\,\{<, \leqslant , \geqslant , >\}$, $x \in \mathbb {Q}_{\geqslant 0}$ and *r* is a reward structure.

As can be seen from the rule for $\theta$, multi-objective properties can incorporate three different types of reward objective in the ${\mathtt R}$ operator and an alternative form of the ${\mathtt P}$ operator. We can reason about *total reward* (indefinitely cumulated rewards; ${\mathtt R}^{r}_{\,\bowtie x}[\,{\mathtt {C}}\,]$), *mean payoff* (long-run average reward; ${\mathtt R}^{r}_{\,\bowtie x}[\,{{\mathtt S}}\,]$), or the long-run *ratio* of two rewards ($\smash {{\mathtt R}^{r_1/r_2}_{\,\bowtie x}[\,{{\mathtt S}}\,]}$). The ${\mathtt P}$ operator is used to specify almost-sure (i.e. probability 1) satisfaction objectives for mean payoff and ratio rewards.

As for the single-objective case, each individual objective is associated with a threshold $\bowtie x$ to be met. Objectives are combined with the standard Boolean connectives. (Only $\lnot$ and $\wedge$ are shown in the grammar, but $\vee$ and $\rightarrow$ can be derived in the usual fashion.) The objectives within a single multi-objective query must be of the same type, and a query comprising almost-sure satisfaction objectives must take the form of a single conjunction. Multi-objective properties focus solely on expected reward properties, but note that expected total reward properties can be used to encode conventional probabilistic reachability.

**Fig. 3:**
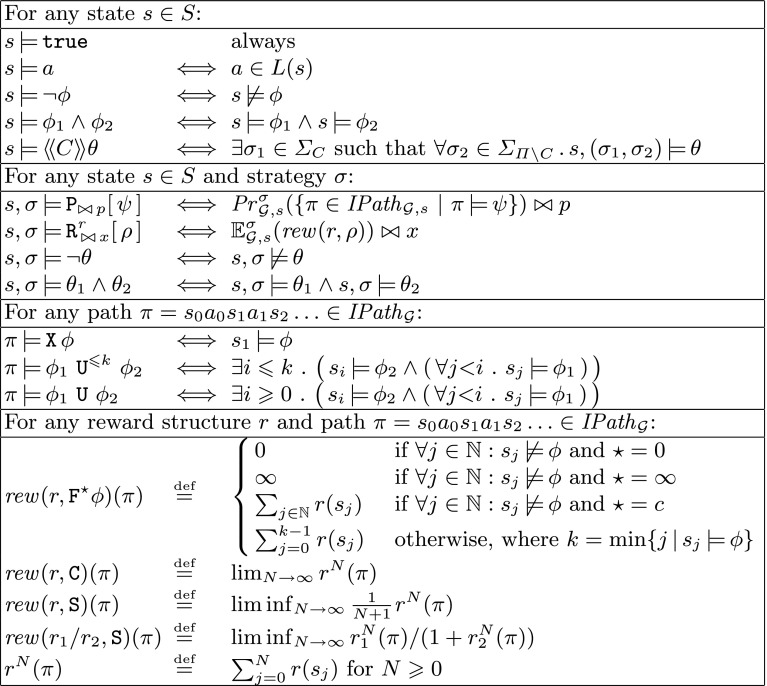
Semantics of the PRISM-games property specification language for an SMG $\mathcal {G}$

Examples of multi-objective queries, again for the robot navigation example of Sect. 2, are as follows:

- $\langle \!\langle \{ robot _1\} \rangle \!\rangle ({\mathtt R}^{ energy _1}_{\,\leqslant e_1}[\,{\mathtt {C}}\,] \; \wedge \; {\mathtt R}^{ tasks _1}_{\,\geqslant t_1}[\,{\mathtt {C}}\,])$ —robot 1 has a strategy which ensures that both the expected total energy consumed is at most $e_1$ and the expected total number of tasks completed is at least $t_1$, regardless of the behaviour of robots 2 and 3.
- $\langle \!\langle \{ robot _1, robot _2\} \rangle \!\rangle ( {\mathtt R}^{ energy_1 / time }_{\,\leqslant e_1}[\,{{\mathtt S}}\,] \; {\vee } \; {\mathtt R}^{ energy_2 / time }_{\,\leqslant e_2}[\,{{\mathtt S}}\,] )$ — robots 1 and 2 have a combined strategy which ensures that either the expected long-run energy consumption rate of robot 1 is at most $e_1$ or it is at most $e_2$ for robot 2, whatever robot 3 does.

For these properties, we assume that additional reward structures $$ energy _i$$ and $$ tasks _i$$ (for $$i=1,2,3$$) are added to the model, which track the energy consumed by robot *i* and the number of tasks that it has completed, respectively. For the purposes of these examples, we also ignore the restrictions imposed by PRISM-games as to whether the SMG is a stopping game or controllable multi-chain game (see Sect. 4.2 for details).

In the same way that single-objective properties can be written in a *numerical* form ($\min =?$ or $\max =?$) to obtain optimal values directly, PRISM-games allows the extraction of Pareto sets to visualise the trade-offs between objectives in a multi-objective query (see Sects. 4, 5 for details).

### Property semantics

We conclude our discussion of the PRISM-games property specification language with a presentation of its formal semantics. A property $\phi$ of an SMG $\mathcal {G}$ (for either of the two grammars presented above) is interpreted for a state *s* of $\mathcal {G}$. We write $s {\,\models \,}\phi$ to denote that $\phi$ is satisfied (i.e. is true) in state *s*. The formal definition of the relation ${\,\models \,}$ is shown in Fig. 3. As can be seen, the definition also uses several auxiliary satisfaction relations. In particular, $s,{\sigma }{\,\models \,}\theta$ means that, when the game is under the control of strategy ${\sigma }$, $\theta$ is satisfied in state *s*. This is used for the definition of the semantics of the coalition operator $\langle \!\langle C \rangle \!\rangle \theta$, which requires the existence of a (combined) strategy ${\sigma }_1$ for all players in *C* such that, for all possible strategies ${\sigma }_2$ of the other players, $s,({\sigma }_1,{\sigma }_2) {\,\models \,}\theta$ holds, where $({\sigma }_1,{\sigma }_2)$ is the strategy combining the individual strategies for all players in the game.

## Algorithms

We now describe the implementation of PRISM-games in more detail. In this section, we summarise the key algorithms required for model checking and give pointers to further information. In the next section, we will describe the overall architecture of the tool and explain some of the low-level implementation details.

The key algorithmic task performed by PRISM-games is model checking of a property expressed in the logic of Sect. 3 against an SMG constructed from a description in the PRISM modelling language. Since the property specification language is based on a branching-time logic (it extends PCTL, which in turn extends CTL), the basic model checking algorithm performs a recursive traversal of the parse tree of the logical formula to be checked and, for each subformula, determines the set of states of the model that satisfy it.

The key operator for model checking is the coalition operator $\langle \!\langle C \rangle \!\rangle \theta$, where $\theta$ is either a single (probabilistic or reward) objective or a Boolean combination of such objectives. In either case, model checking essentially reduces to a strategy synthesis problem: determining whether the set of players *C* has a combined strategy that achieves the objective (or objectives), irrespective of the strategies of the other players.

This problem always reduces to a strategy synthesis problem on a (turn-based) *stochastic two-player game*, in which the first player represents the players in *C* and the second player represents the others (a so-called *coalition game*). Thus, the main component of the SMG model checking algorithm is strategy synthesis for either single- or multi-objective queries on a stochastic two-player game.

### Single-objective strategy synthesis

For single-objective strategy synthesis, determining if a suitable strategy exists in this game reduces to computing *optimal* strategies for player 1, i.e. strategies that minimise or maximise the value of the objective. The problems of computing optimal probabilities of reaching a set of states or expected cumulative rewards in stochastic two-player games are known to be in the complexity class NP $\cap$ coNP [18]. However, in practice, we can use *value iteration* algorithms, which iteratively approximate the optimal values for all states of the model, until some specified convergence criterion is met. This approach can be adapted to handle each of the variants of the ${{{\mathtt {F}}}\ }^{\!\!\star }$ reward operators described in Sect. 3.1. This relies on the use of graph-based precomputation algorithms (e.g. to identify states from which the expected reward is infinite) and, for the case $\star =0$, the use of two successive instances of value iteration which compute an upper and lower bound on the required values, respectively. The process is described in more detail in [12].

For the cases of probabilistic reachability or until formulas (${\mathtt P}_{\bowtie \,p}[\,{{{{\mathtt {F}}}\ }\phi }\,]$ or ${\mathtt P}_{\bowtie \,p}[\,{\phi _1{\ {\mathtt {U}}\ }\phi _2}\,]$) and cumulative expected reward (${\mathtt R}^{r}_{\,\bowtie \,x}[\,{{{{\mathtt {F}}}\ }\!\!^{\star }\phi }\,]$), PRISM-games synthesises optimal strategies that are both memoryless and deterministic. For the bounded variants of reachability/until, a finite-memory strategy is generated. In each case, the optimal strategy can be inspected in the tool, or exported to a file for further analysis.

### Multi-objective strategy synthesis

For *multi-objective* properties (which, again, only need to be considered on a stochastic *two-player* game), PRISM-games implements the techniques presented in [5, 7, 14, 16]. The syntax described in Sect. 3.2 allows a variety of different reward objectives to be used: expected total reward, expected long-run average reward (mean payoff), expected long-run reward ratio and almost-sure (probability 1) variants of the latter two.

For the purposes of model checking, PRISM-games imposes some restrictions on the classes of SMGs for which these can be checked. For expected total rewards, games must be *stopping* and, for expected long-run objectives, games must be *controllable multi-chain* (see Sect. 2.2 for details of these subclasses).

**Fig. 4:**
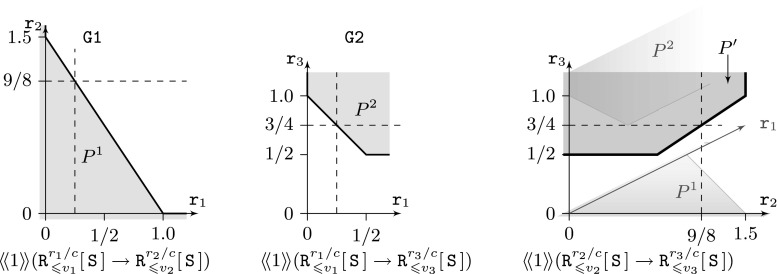
Pareto sets generated by PRISM-games for compositional strategy synthesis (see Sect. 4.3)

At the heart of the strategy, synthesis algorithm is a unified fixpoint computation, used for all classes of properties after appropriate transformations have been applied. In particular, Boolean combinations of expectation objectives are converted to conjunctions by selecting appropriate *weights* for the individual objectives (see [14, Theorem 6] for details).

The core task is then to compute the set of *achievable* values for a conjunction of expected reward objectives. Assuming a conjunction of *n* reward objectives, with thresholds $x_1,\dots ,x_n$, a vector of *n* values is said to be achievable if a single (player 1) strategy of the game can simultaneously satisfy the threshold $x_i$ for each of the *n* reward objectives.

Strategy synthesis is again performed using a value iteration style algorithm, but now computing, for each state *s* of the model, a *polytope* $P_S\subseteq \mathbb {R}^n$ of achievable values for that state, rather than a single value as in the single-objective case. In fact, the value iteration algorithm computes and stores the *Pareto set* of optimal values for the polytope, i.e. the frontier set containing achievable values that cannot be improved in any direction without degrading another. The full polytope is represented by its downward closure.

Since value iteration performs an approximate computation, up to a specified accuracy, the algorithm in fact constructs an $\varepsilon$-approximation of the Pareto set. Improvements in performance can be achieved by computing successive polytopes using in-place (i.e. Gauss–Seidel) updates, as well as rounding the corners of the polytopes at every iteration (where the latter comes at the cost of precision) [16].

From the results of the value iteration computation, a succinct *stochastic memory update* strategy (see Sect. 2.1 for the definition) can be constructed, which achieves the required values. Intuitively, the vertices of the polytope for each state form the memory elements of the constructed strategy and are used to track the values that need to be achieved for each objective. As for multi-objective strategy synthesis on the simpler model of MDPs [23], randomisation is needed in the strategy to capture trade-offs between objectives. See [16, 44] for more information and some detailed examples.

**Fig. 5:**
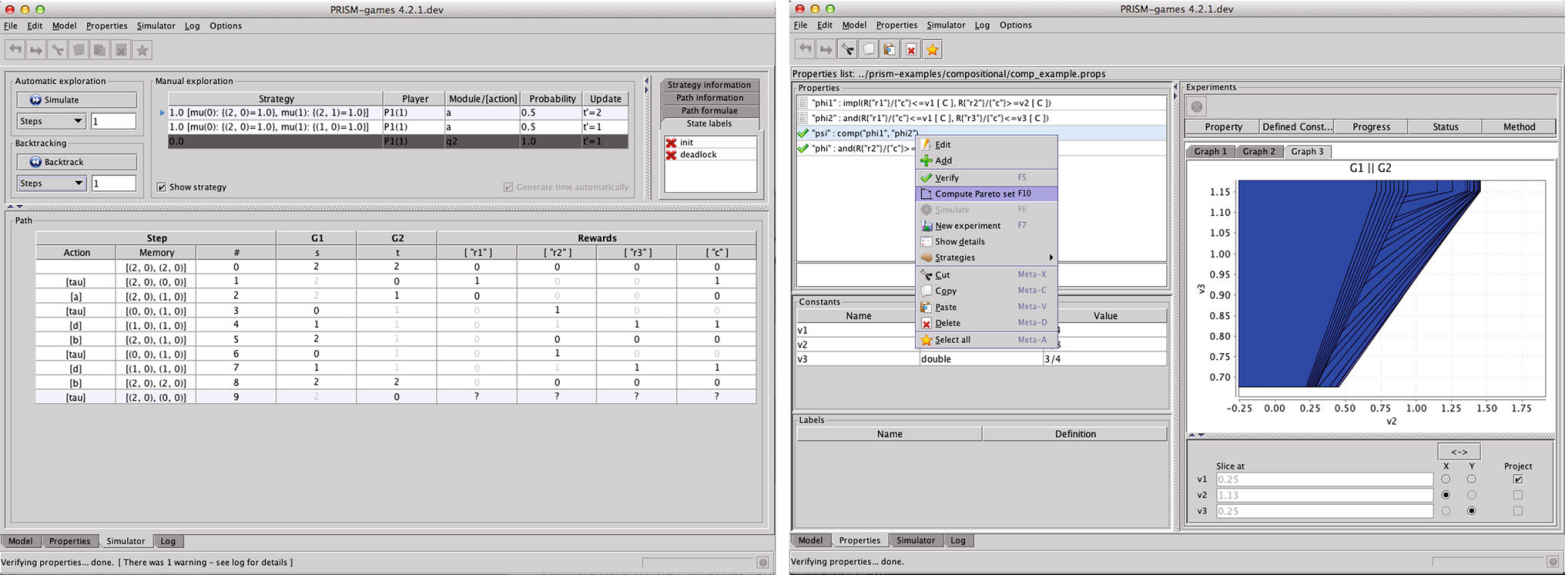
Screenshots of the PRISM-games graphical user interface. Left: a synthesised strategy for an SMG being manually explored in the simulator. Right: visualisation of a Pareto set from multi-objective strategy synthesis (here, a property with three objectives is displayed graphically by projecting the Pareto set onto the second and third objectives)

In a similar fashion to the handling of numerical queries for single-objective properties, PRISM-games can also provide direct access to the Pareto set for a multi-objective property. In practice, these are visualised by selecting two-dimensional *slices* of the full set (see Sect. 5 and Fig. 5 for an example).

### Compositional strategy synthesis

Finally, we discuss the functionality in PRISM-games for *compositional* strategy synthesis techniques. Building upon assume-guarantee verification rules for probabilistic automata (i.e. games with only a single player), proposed in [33], support is provided for assume-guarantee strategy synthesis in stochastic two-player games [6, 7].

Given a system $\mathcal {G}$ composed of subsystems $\mathcal {G}_1$, $\mathcal {G}_2$, $\ldots$, a designer supplies respective *local property specifications* $\varphi _1$, $\varphi _2$, $\ldots$ via the construct comp( $\varphi _1$ , $\varphi _2$ , ...). By synthesising *local strategies* ${\sigma }_i$ for $\mathcal {G}_i$ satisfying $\varphi _i$, a *global strategy* ${\sigma }$ can be constructed for $\mathcal {G}$. Using *assume-guarantee rules*, one can then derive a *global property* $\varphi$ for $\mathcal {G}$ that is satisfied by ${\sigma }$. The rules require *fairness* conditions, and we write $\mathcal {G},{\sigma }\models ^{\textsf {u}} \varphi$ if the player 1 strategy ${\sigma }$ satisfies $\varphi$ against all unconditionally fair player 2 strategies. For example, the rule:states that player 1 wins with strategy  for $\varphi ^G$ in the top-level system if ${\sigma }_2$ in $\mathcal {G}_2$ achieves $\varphi ^G$ under the contract $\varphi ^A \rightarrow \varphi ^G$, and ${\sigma }_1$ in $\mathcal {G}_1$ satisfies $\varphi ^A$.

The tool compositionally computes a Pareto set for the property $\varphi$ of the top-level system, which is an under-approximation of the Pareto set computed directly on the monolithic system. For a target in the compositional Pareto set, the targets for the local property specifications $\varphi _i$ can be derived, so that the local strategies can be synthesised. Figure 4 shows an example, with the property specifications given beneath each of the generated Pareto sets, using ratio reward objectives and 4 reward structures $r_1$, $r_2$, $r_3$ and *c*. The rightmost image shows the compositional Pareto set $P'$. The global target is $({\texttt {v}}_2,{\texttt {v}}_3) = (\frac{3}{4},\frac{9}{8})$, and the local targets can be seen to be consistent with ${\texttt {v}}_1 = \frac{1}{4}$.

## Architecture and implementation

PRISM-games is implemented primarily in Java. The tool is open source, currently released under the GPL, and is available from the PRISM-games website:

http://www.prismmodelchecker.org/games/

which also includes documentation, examples and related publications, as well as links to resources shared with PRISM, such as the support and discussion forums. The source code can also be accessed and browsed from the PRISM project’s GitHub page:

https://github.com/prismmodelchecker

This site also hosts resources such as the benchmark and regression test suites, which are currently being extended with SMG model checking examples.

### Architecture

PRISM-games is an extension of PRISM and so shares and extends the same basic architecture as well as various components from the original tool. This includes:

- *Parsers* for the modelling language and property specification languages, which are extended to support games and the new strategy synthesis properties.
- The *discrete-event simulator*, used for manual or automatic model exploration, which now supports SMGs, as well as the new notions of two-player parallel composition for assume-guarantee strategy synthesis.

Like its parent tool, the functionality within PRISM-games can be accessed in a variety of ways:

- The *command-line* interface, which is well suited for automated verification runs, long-running tasks or remote invocation.
- The *graphical user interface* (GUI), which provides editors for models and properties, graph plotting functionality and a simulator. Notable new features in the GUI include the ability to explore synthesised game strategies in the simulator, the possibility to apply a generated strategy to a game model and then perform further model checking, and the ability to visualise Pareto sets from multi-objective strategy synthesis. Screenshots illustrating the first and third of these can be found in Fig. 5.
- The *application programming interface (API)*, which provides direct programmatic access (in Java) to the underlying model checking engine used by both the command-line and graphical user interfaces.

### Data structures

We now give some details of the key data structures needed to implement model checking of SMGs and the operations needed to manipulate them. Further details can be found in [44].

**Games and strategies** The current implementation of model checking within PRISM-games uses explicit-state data structures, building upon PRISM’s existing “explicit” engine which is written in Java.

Stochastic multi-player games are represented using sparse matrix data structures. For the purposes of performing strategy synthesis (which is primarily based on value iteration), the sparse matrices are equipped with various matrix-vector multiplication algorithms, which form the most expensive part of the solution process.

In order to support compositional modelling and verification, PRISM-games adds support to the data structures for combining SMGs using the notion of parallel composition from [6], described in Sect. 2.3. This includes various auxiliary steps, including an (optional) compatibility check and normal form transformation.

Strategies are also implemented in an explicit-state fashion. Memoryless strategies are represented as vectors and stochastic memory update strategies as maps encoding the next choice and memory update functions. For compositional verification within the assume-guarantee framework, the synthesised strategies are not explicitly composed, but the individual strategies are stored separately. When simulating a composed game under a composed strategy, the memory update is performed at each step only for the strategies corresponding to the involved components.

**Polytopes** For single-objective SMG model checking, the core numerical computation techniques compute a single value (e.g. a probability or expected reward value) for each state of the SMG. These are mostly performed using fixpoint approximations over state-indexed vectors of floating point numbers. However, for multi-objective model checking, we need to compute sets of achievable objective values for each state, which can be done by associating each state with a convex, closed polytope.

Polytope representation and manipulation is done using the Parma Polyhedra Library (PPL) [4]. In particular, this provides support for performing the set-theoretic operations of convex union and intersection. The PPL library represents each polytope by both its vertices and the set of bounding hyperplanes, called the Motzkin double description method [4]. The vertex representation also allows for *rays*, which we use for downward closure operations: a ray is a vector $\mathbf {y}$ such that, for any point $\mathbf {x}$ in a polytope *X*, any point $\mathbf {x} + \alpha \cdot \mathbf {y}$ is also in *X*, for any $\alpha > 0$.

Both representations are equally expressive, but differ in how efficient operations are performed: intersection of polytopes is more efficient using hyperplanes (by taking the union of the bounding hyperplanes); convex union is more efficient using the vertices (by taking the union of the vertices). PPL automatically performs transformations between the representations and can minimise the representation size. This representation of the polytopes is symbolic in the sense that we represent a polytope, an infinite set of points, by a finite set of vertices and hyperplanes.

**Fig. 6:**
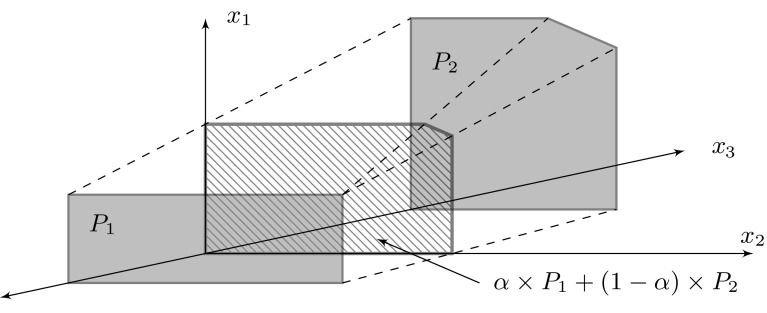
Illustrating the computation of the weighted Minkowski sum. $P_1$ is shifted from the origin by $x_3 = -\frac{1}{1-\alpha }$, $P_2$ is shifted from the origin by $x_3 = \frac{1}{\alpha }$

**Weighted Minkowski sum** One operation required by PRISM-games for manipulating polytopes, but not directly supported by the PPL library, is the *weighted Minkowski sum*. This is one of the core operations used during the iterative approximation of Pareto sets for multi-objective strategy synthesis, with the weights corresponding to the probabilities attached to outgoing transitions from each state of the SMG. Given sets $X, Y \subseteq \mathbb {R}^n$, their Minkowski sum is the set ; given also a weight $\alpha \in [0,1]$ their weighted Minkowski sum is the set .

We implement this operation using PPL’s vertex representation, which we explain here for two polytopes $P_1$ and $P_2$. Let  for $i \in \{1, 2\}$, where $V_i$ is an $n \times m_i$ matrix defining the vertices of the polytope. The idea behind computing their weighted Minkowski sum is the following. First, lift the space to dimension $n+1$ and place $P_1$ and $P_2$ at a distance of $-\frac{1}{1-\alpha }$ and $\frac{1}{\alpha }$ from the origin, respectively. Their convex hull, shown with dashed lines in Fig. 6, can then be computed as:

$$\begin{aligned}&\left\{ \mathbf {x} \in \mathbb {R}^{n+1} \,|\, \exists \mathbf {y} \in \mathbb {R}^{m_1 + m_2 + 1} \cdot \right. \\&\left. \quad \begin{bmatrix} V_1&V_2 \\ \mathbf {1}^T/\alpha&-\mathbf {1}^T/(1-\alpha ) \end{bmatrix} \mathbf {y} = \mathbf {x} \wedge \mathbf {1}^T\mathbf {y} = 1 \right\} . \end{aligned}$$

Constraining this polytope to $x_{n+1} = 0$, we get $\mathbf {1}^T\mathbf {y}_1 = \alpha$ and $\mathbf {1}^1\mathbf {y}_2 = 1 - \alpha$, and hence we define $\alpha \mathbf {z}_1 = \mathbf {y}_1$ and $(1-\alpha )\mathbf {z}_2 = \mathbf {y}_2$, so that $\mathbf {x} = \alpha V_1 \mathbf {z}_1 + (1-\alpha ) V_2 \mathbf {z}_2$ and $\mathbf {1}^T\mathbf {z}_1 = \mathbf {1}^T\mathbf {z}_2 = 1$. This corresponds to computing the weighted Minkowski sum $\alpha \times P_1 + (1-\alpha ) \times P_2$, as illustrated by the hatched polytope in Fig. 6. Computing the convex hull and constraining to $x_{n+1} = 0$ are supported by PPL. This method extends to more than two polytopes in a similar fashion by introducing an extra dimension per polytope [30]. For more details and examples of the operation, see [44, Sec.s 6.2 and 7.2].

Note that the weighted Minkowski sum is the most computationally expensive operation that we have to implement, as the number of vertices of the polytope $\alpha P_1 + (1-\alpha ) P_2$ is $\mathcal {O}(|P_1| \cdot |P_2|)$; see Theorem 4.1.1 of [43]. In contrast, the number of vertices of the polytopes $P_1 \cap P_2$ and $\textsf {conv}(P_1 \cup P_2)$ is $\mathcal {O}(|P_1| + |P_2|)$ in both cases. The performance of our synthesis algorithms is therefore dependent on the outgoing branching degree of the states.

## Experimental results

Next, we present some experimental results for a selection of models analysed with PRISM-games, in order to give an illustration of the scalability of the tool. Although the property specification language presented in Sect. 3 allows multiple coalition operators $\langle \!\langle C \rangle \!\rangle \theta$ to be combined, either using Boolean combinations or nesting of subformulas, in practice, most queries are single instances of the operator so we restrict our attention to such properties. We discuss the cases of single-objective and multi-objective queries separately, since the techniques used to check them are quite different.

First, Table 1 shows statistics for a selection of models and the time taken to check various single-objective properties. The models are taken from three case studies: *team-form* (team formation protocols) [15], *mdsm* (microgrid demand-side management) [12, 38] and *investor* (future markets investor) [35]. Each case study has a parameter that can be varied to yield increasingly large models. (Details can be found in the references cited above.) The table shows the number of players and model size (number of states) for each one.

Again, we focus our attention on the kinds of objectives used most frequently in practice, which refer to either the probability of reaching a set of target states, or the expected reward accumulated before reaching it. For the latter, we show two of the three possible variants. Times are presented for performing strategy synthesis in each case using a 2.80GHz PC with 32GB RAM. Since this process reduces to the analysis of a stochastic two-player game, the number of players in the original SMG has no impact on the solution time. Instead, the model size is the key factor. We observe that, for a given example, the time for strategy synthesis increases roughly linearly with the increase in state space size. This is as expected since these properties are checked using an iterative numerical method, each iteration of which performs an operation for each state of the model.

PRISM-games is able to work with games of up to approximately 6 million states in these examples, which is comparable to the situation for similar models such as MDPs when using the “explicit” model checking engine on which PRISM-games is based. Future work will adapt PRISM’s symbolic model checking engines, which can provide increased scalability in some cases, to SMGs.

**Table 1:** A selection of experimental results for single-objective strategy synthesis queries

Case study [parameters]		SMG statistics		Strategy synthesis	
		Players	States	Property type	Time (s)
*team-form* [*N*]	3	3	12,475	$\langle \!\langle C \rangle \!\rangle {\mathtt P}_{\max =?}[\,{{{{\mathtt {F}}}\ }\phi }\,]$	0.2
	4	4	96,665		0.9
	5	5	907,993		11
*mdsm* [*N*]	5	5	743,904	$\langle \!\langle C \rangle \!\rangle {\mathtt R}^{\max =?}_{\,r}[\,{{{{\mathtt {F}}}\ }\!\!^{\infty } \phi }\,]$	62
	6	6	2,384,369		222
	7	7	6,241,312		1055
*investor* [*vmax*]	10	2	10,868	$\langle \!\langle C \rangle \!\rangle {\mathtt R}^{\max =?}_{\,r}[\,{{{{\mathtt {F}}}\ }\!\!^{c} \phi }\,]$	0.7
	100	2	750,893		122
	200	2	2,931,643		821

**Table 2:** A selection of experimental results for multi-objective/compositional strategy synthesis queries

Case study	SMG statistics		Strategy synthesis			
	Components	States	Num. obj.s	Objective types	Accuracy	Time (s)
*uav*	1	6,251	2	Exp. total, Pareto	0.1	652
*uav*	1	6,251	2	Exp. total, Pareto	0.01	871
*driving [charlton]*	1	501	3	Exp. total	0.001	2603
*driving [islip]*	1	1,527	3	Exp. total	0.001	1968
*power [d=0]*	2	7,296/7,296	3/3	Almost-sure ratio	0.01	586/484
*power [d=1]*	2	24,744/24,744	3/3	Almost-sure ratio	0.01	3325/2377
*temp*	3	1,478/1,740/1,478	3/2/3	Exp. ratio	0.05	829/69/734
*temp*	3	1,478/1,740/1,478	3/2/3	Exp. ratio	0.01	860/92/2480

Table 2 shows statistics for multi-objective strategy synthesis. The models are taken from four case studies: *uav* (human-in-the-loop UAV mission planning) [24], *driving* (autonomous urban driving) [16], *power* (aircraft power distribution) [5] and *temp* (temperature control) [44]; the first three are discussed in the next section.

The case studies do not all have parameters to scale the models, as in Table 1, but we show two variants of *driving* (using maps for two villages), and for *power*, we vary the switch delays *d*. The last two case studies are used for compositional (assume-guarantee) strategy synthesis. We do not focus on that aspect here, but simply use it as a source of multi-objective queries. The table shows the number of components (subsystems), and sizes/times are given for each one, separated by slashes in the table entries. We omit the number of players since this is 2 in all cases.

The final column shows the time required for strategy synthesis, using the same hardware as above. In addition to considering different models, we also vary the accuracy with which Pareto curves are approximated. For multi-objective strategy synthesis, we observe that performance depends on multiple factors. Again, model size affects the time (see, e.g. the *power* example), but the number of objectives also has a significant impact (see, e.g. the *temp* example) and increasing the solution accuracy also results in longer runtimes.

## Case studies

PRISM-games has been under development since 2012 and has since then been successfully deployed to model and analyse systems across a wide range of application domains. In this section, we survey some representative examples. Further details of several of the examples can be found in [38, 40, 42, 44], and an up-to-date list of case studies is maintained at the tool web site [45].

**Microgrid demand-side management** [38] The example models a decentralised energy management protocol for smart grids that draw energy from a variety of sources. The system consists of a set of households, where each household follows a simple probabilistic protocol to execute a load if the current energy cost is below a pre-agreed limit, and otherwise it only executes the load with a pre-agreed probability. The energy cost to execute a load for a single time unit is the number of loads currently being executed in the grid. The analysis of the protocol with respect to the expected load per cost unit for a household, formulated as a single-objective total reward property, exposed a protocol weakness. The weakness was then corrected by disincentivising non-cooperative behaviour.

**Human-in-the-loop UAV mission planning** [24] This case study concerns autonomous unmanned aerial vehicles (UAV) performing road network surveillance and reacting to inputs from a human operator. The UAV performs most of the piloting functions, such as selecting the waypoints and flying the route. The human operator performs tasks such as steering the onboard sensor to capture imagery of targets, but may also pick a road for the UAV at waypoints. The optimal UAV piloting strategy depends on mission objectives, e.g. safety, reachability, coverage and operator characteristics, i.e. workload, proficiency and fatigue. The main focus of the case study is on studying a multi-objective property to analyse the trade-off between the mission completion time and the number of visits to restricted operating zones, which have been investigated by computing Pareto sets.

**Autonomous urban driving** [16] An SMG model of an autonomous car is developed, which considers the car driving through an urban environment and reacting to hazards such as pedestrians, obstacles and traffic jams. The car not only decides on the reactions to hazards, which are adversarial, but also chooses the roads to take in order to reach a target location. The presence and probability of hazards are based on statistical information for the road. Through multi-objective strategy synthesis, strategies with optimal trade-off between the probability of reaching the target location, the probability of avoiding accidents and the overall quality of roads on the route are identified.

**Aircraft power distribution** [5] An aircraft electrical power network is considered, where power is to be routed from generators to buses through controllable switches. The generators can exhibit failures and switches have delays. The system consists of several components, each containing buses and generators, and the components can deliver power to each other. The network is modelled as a composition of stochastic games, one for each component. These components are physically separated for reliability and hence allow limited interaction and communication. Compositional strategy synthesis is applied to find strategies with good trade-off between uptime of buses and failure rate. By employing stochasticity, we can faithfully encode the reliability specifications in quantitative fashion, thus improving over previous results. The property is modelled as a conjunction of ratio reward properties.

**Self-adaptive software architectures** [10, 26] Self-adaptive software automatically adapts its structure and behaviour according to changing requirements and quantitative goals. Several self-adaptive software architectures, such as adaptive industrial middleware used to monitor and manage sensor networks in renewable energy production plants, have been modelled as stochastic games and analysed. Both single- and multi-objective verification of multi-player stochastic games has been applied to evaluate their resilience properties and synthesise proactive adaptation policies.

**DNS bandwidth amplification attack** [22] The Domain Name System (DNS) is an Internet-wide hierarchical naming system for assigning IP addresses to domain names, and any disruption of the service can lead to serious consequences. A notable threat to DNS, namely the bandwidth amplification attack, where an attacker attempts to flood a victim DNS server with malicious traffic, is modelled as a stochastic game. Verification and strategy synthesis is used to analyse and generate countermeasures to defend against the attack.

**Attack–defence scenarios in RFID goods management system** [3] This case study considers complex attack–defence scenarios, such as an RFID goods management system, translating attack–defence trees to two-player stochastic games. Probabilistic verification is then employed to check security properties of the attack–defence scenarios and to synthesise strategies for attackers or defenders which guarantee or optimise some quantitative property. The properties considered include single-objective properties such as the probability of a successful attack or the incurred cost, as well as their multi-objective combinations.

## Related tools

There are a variety of probabilistic model checking tools currently available. PRISM-games builds upon components of the PRISM [32] tool, as discussed in Sect. 5. Other general-purpose probabilistic model checkers include MRMC [31], STORM [21], the Modest Toolset [29], iscasMc [27] and PAT [39]. However, none of these provide support for stochastic games.

Other tools exist for the analysis of game models, but have a different focus to PRISM-games. For stochastic games, there is support for qualitative verification in GIST [11] and partial support in the general-purpose game solver GAVS+ [17], but there are no tools for multi-objective or compositional analysis.

Analysis of Nash equilibria can be performed with EAGLE [41] or PRALINE [9], but only for non-stochastic games. Lastly, Uppaal Stratego [19] performs strategy synthesis against quantitative properties, but with a focus on real-time systems.

We also mention that multi-objective probabilistic verification, one of the key features of PRISM-games, is also available elsewhere for simpler models, notably Markov decision processes. This is supported by general-purpose model checkers, such as PRISM [32] and STORM [21], and the more specialised tool MultiGain [8].

## Conclusions and future work

PRISM-games is a tool for verification and strategy synthesis of stochastic multi-player games. It incorporates an array of techniques to support the generation of strategies specified by a wide range of formally specified quantitative properties, including single-objective and multi-objective variants. In this paper, we have provided an overview of the tool, from both a user perspective and in terms of the underlying implementation.

Various extensions are under development or planned for the future. Firstly, support for a wider range of temporal properties, specified in linear temporal logic (LTL), is in progress. Secondly, symbolic implementations of model checking are being added, to complement the current explicit-state version. Initially, this builds upon the existing symbolic techniques implemented in PRISM for other probabilistic models using binary decision diagrams (BDDs) and multi-terminal BDDs.

Future work will investigate verification and strategy synthesis techniques for alternative game-theoretic solution concepts such as Nash equilibria and wider classes of stochastic games, such as concurrent variants and games operating under partial observability.
